# Perceptions and future perspectives of medical students on the use of artificial intelligence based chatbots: an exploratory analysis

**DOI:** 10.3389/fmed.2025.1529305

**Published:** 2025-01-22

**Authors:** Juan José Gualda-Gea, Lourdes Estefanía Barón-Miras, Maria Jesús Bertran, Anna Vilella, Isabel Torá-Rocamora, Andres Prat

**Affiliations:** ^1^Department of Preventive Medicine and Epidemiology, Hospital Clínic of Barcelona, Barcelona, Spain; ^2^Barcelona Institute for Global Health (ISGlobal), Barcelona, Spain; ^3^Department of Medicine, Faculty of Medicine, University of Barcelona, Barcelona, Spain

**Keywords:** medical education, artificial intelligence, chatbots, medical students, teaching improvement

## Abstract

**Background:**

Artificial Intelligence (AI) has made a strong entrance into different fields such as healthcare, but currently, medical degree curricula are not adapted to the changes that adopting these types of tools entitles. It is important to understand the future needs of students to provide the most comprehensive education possible.

**Objective:**

The aim of this teaching improvement project is to describe the knowledge, attitudes, and perspectives of medical students regarding the application of AI and chatbots with patients, also considering their ethical perceptions.

**Methods:**

Descriptive cross-sectional analysis in which the participants were students enrolled in the subject “Preventive Medicine, Public Health and Applied Statistics” during the second semester of the 2023/24 academic year, corresponding to the fifth year of the Degree in Medicine at the University of Barcelona. The students were invited to complete a specific questionnaire anonymously and voluntarily, which they could respond to using their mobile devices by scanning a QR code projected on the classroom screen, we used Microsoft Forms to perform the survey.

**Results:**

Out of the 61 students enrolled in the subject, 34 (56%) attended the seminar, of whom 29 (85%) completed the questionnaire correctly. Of those completing the questionnaire, 20 (69%) had never used chatbots for medical information, 19 (66%) expressed a strong interest in the practical applications of AI in medicine, 14 (48%) indicated elevated concern about the ethical aspects, 17 (59%) acknowledged potential biases in these tools, and 17 (59%) expressed at least moderate confidence in chatbot-provided information. Notably, 24 (83%) agreed that acquiring AI-related knowledge will be essential to effectively perform their future professional roles.

**Conclusion:**

Surveyed medical students demonstrated limited exposure to AI-based tools and showed a mid-level of awareness about ethical concerns, but they recognized the importance of AI knowledge for their careers, emphasizing the need for AI integration in medical education.

## 1 Introduction

Digital transformation in the healthcare sector is driving a deep reconfiguration of medical practice, with Artificial Intelligence (AI) emerging as a key factor in addressing current and future healthcare challenges (1). AI-based tools, such as machine learning algorithms and large-scale data analysis, have already demonstrated their capacity to improve diagnostic accuracy and accelerate the early identification of diseases, resulting in more timely interventions and more favorable patient outcomes (2, 3). Additionally, the increasing digitization of information and the incorporation of decision support systems optimize workflows, reduce administrative burdens, and facilitate access to care, even in resource-limited settings (4).

Within this technological ecosystem, chatbots AI-driven conversational assistants have positioned themselves as promising tools to enhance interaction between healthcare professionals and patients (5, 6). These systems can provide immediate responses to basic inquiries, offer reliable information on symptoms and treatments, and promote health education, thereby expanding access to healthcare services beyond geographical and temporal limitations (7, 8). However, the implementation of these technologies is not without challenges, particularly concerning ethical issues and the quality of information provided (9).

AI in healthcare presents ethical dilemmas that encompass information integrity, data privacy, and accountability in algorithm-mediated clinical scenarios (10–12). Furthermore, the potential for biases, informational “hallucinations” (responses that appear valid but are unfounded), and the possible erosion of the doctor-patient relationship underscore the need to address these technologies with prudence and rigor (13–15). In this regard, medical education plays a central role: preparing future healthcare professionals to understand, adopt, and critically evaluate AI tools is essential to ensure their ethical, effective, and patient-centered integration into clinical practice (16, 17).

Although various studies have explored the general perceptions of students and healthcare professionals regarding AI, there remains a gap in the literature concerning the specific understanding that advanced medical students have about the use of chatbots in clinical settings (18). This population is at a critical juncture: on the brink of entering professional practice, their perceptions, concerns, and expectations provide valuable insights into how curricula and training strategies should be shaped to meet the demands of an imminent future marked by the gradual inclusion of AI in healthcare delivery (11, 14, 18, 19). Understanding their attitudes, knowledge levels, and ethical concerns offers a solid foundation for designing curricula that balance technical training with ethical reflection, promoting responsible and informed use of AI.

In this context, the present teaching improvement project aims to describe the knowledge, attitudes, and perspectives of medical students regarding the application of AI and the use of chatbots in the healthcare field, with particular attention to their ethical perceptions. This approach seeks to generate an initial framework to guide the future inclusion of AI-related content in medical education, ensuring that tomorrow’s physicians are better prepared to integrate these tools into their clinical practice competently and ethically.

## 2 Materials and methods

### 2.1 Study design

A descriptive cross-sectional study was conducted with the aim of obtaining an initial understanding of medical students’ perceptions and attitudes regarding the integration of AI-based chatbots in the healthcare sector.

### 2.2 Population and sampling

The target population comprised students enrolled in the course “Preventive Medicine, Public Health, and Applied Statistics,” corresponding to the fifth year of the Medicine Degree at the University of Barcelona, during the second semester of the 2023/24 academic year. A non-probabilistic sampling method was employed, selecting participants who attended a theoretical seminar on the use of chatbots in the medical field and who voluntarily agreed to complete the questionnaire.

### 2.3 Sample size

The sample size was determined by seminar attendance and voluntary participation in the survey. Given the exploratory and preliminary nature of the study, an ideal sample size was not calculated using specific statistical formulas. The sample included students who, prior to the seminar, scanned a QR code and completed the online questionnaire using the Microsoft Forms application.

### 2.4 Instrument development

A quantitative questionnaire was designed, structured into three main sections with a total of 14 items. The questionnaire was intended to be simple, providing an initial approximation of students’ perceptions. Each question featured a closed-response format (predefined options or Likert scales ranging from 1 to 5). The three dimensions investigated were:

- Attitudes and Prior Knowledge (3 items): Assesses previous familiarity with AI tools and chatbots in medicine.

- Ethical Perceptions (3 items): Explores ethical concerns and the level of trust in information provided by chatbots.

- Future Perspectives (8 items): Investigates the future relevance of AI knowledge for medical practice and professional training.

Q1. Have you ever used chatbots to obtain medical information? 1-Never, 5-Frequently.

Q2. Are you familiar with current artificial intelligence tools applied to medicine, such as AI-assisted diagnosis or therapeutic recommendations? 1 - not at all, 5 - very much.

Q11. I am interested in the practical aspects of AI in medicine. 1-very little, 5-a lot.

Q8. Are you concerned about the ethics of using chatbots in medicine? 1 “not very concerned” and 5 “very concerned.”

Q9. On a scale from 1 to 5, where 1 is “not very confident” and 5 is “very confident,” how much trust do you have in the information provided by chatbots on medical topics?

Q10. Are you concerned about potential biases in such tools? 1-very little, 5-a lot.

Q3. The use of AI in healthcare can positively change medicine. 1-Strongly disagree, 5-Strongly agree.

Q4. The use of AI can negatively affect the doctor-patient relationship. 1-Strongly disagree, 5-Strongly agree.

Q5. Doctors will need to know about AI-based tools to perform their jobs in the near future. 1-Strongly disagree, 5-Strongly agree.

Q6. AI should be part of medical education. 1-Strongly disagree, 5-Strongly agree.

Q7. Practical content on the use of AI-based tools in medicine should be introduced in medical degree programs. 1-Strongly disagree, 5-Strongly agree.

Q12. The use of such tools will lead to a dehumanization of medicine. 1-Strongly disagree, 5-Strongly agree.

Q13. The use of such tools will create dependency among medical staff. 1-Strongly disagree, 5-Strongly agree.

Q14. The imposition of these new technologies may influence the choice of specialization for medical personnel. 1-Strongly disagree, 5-Strongly agree.

This instrument was applied in its second iteration, following a pilot test conducted with 14 students in a workshop. This pilot allowed for the adjustment and consensus of questions with expert faculty members to enhance clarity and relevance.

### 2.5 Reliability and validity

Given the preliminary and exploratory nature of the study, comprehensive psychometric analyses (e.g., formal internal reliability tests or construct validity assessments) were not performed. However, the questionnaire underwent review by expert faculty in the fields of preventive medicine and public health, as well as medical education, to ensure clarity, internal consistency, and item relevance. Future research is recommended to formally validate the instrument, including conducting more extensive pilot tests and appropriate psychometric analyses to strengthen the questionnaire’s reliability and validity.

### 2.6 Data collection procedure

Prior to the commencement of the theoretical seminar, students were invited to complete the questionnaire anonymously and voluntarily. Participation involved scanning a QR code projected in the classroom and responding to the questionnaire on their personal mobile devices via Microsoft Forms. To prevent duplicate responses, a time limit was set for completing the questionnaire. No personal, health-related, or sensitive data were collected. Participants were informed about the confidentiality of their responses and their right to abstain from answering or to withdraw from the survey at any time without any consequences.

### 2.7 Data analysis plan

Data analysis was structured according to the three sections of the questionnaire: Attitudes and Prior Knowledge, Ethical Perceptions, and Future Perspectives. The following techniques were employed:

#### 2.7.1 Descriptive statistics

Relative frequency calculations were utilized to characterize responses within each section of the questionnaire. This provided a quantitative overview of participants’ knowledge and opinions on AI and chatbots prior to their exposure to the theoretical seminar.

#### 2.7.2 Visual analysis using horizontal bar charts

Horizontal bar charts were employed to graphically represent the results, facilitating visual comparison of response distributions on a scale of 1 to 5. This type of visualization aids in quickly identifying trends and patterns within the collected data.

#### 2.7.3 Integrated findings summary

Results from each section were synthesized to present a comprehensive conclusion, analysing medical students’ perspectives on the integration of AI-based chatbots in healthcare. This approach prioritized quantitative aspects, allowing for a deeper exploration of participants’ views beyond numerical data.

As an exploratory study, complex inferential methods or systematic evidence synthesis were not employed, limiting the analysis to basic quantitative description and the identification of general patterns in students’ perceptions.

## 3 Results

A total of 61 students were enrolled in the course “Preventive Medicine, Public Health, and Applied Statistics” during the second semester of the 2023/24 academic year. Of these, 34 (56%) students attended the theoretical seminar on the use of chatbots in the medical field, and 29 (85%) of them fully completed the questionnaire. The results are organized according to the three dimensions outlined in the study’s objective: initial knowledge and attitudes, ethical perceptions, and future perspectives on the integration of AI in clinical practice.

### 3.1 Attitudes and prior knowledge

This dimension aimed to describe the initial level of familiarity with AI tools and chatbots, as well as the interest in their application. The results indicated a low degree of prior exposure to these technologies:

Previous Use of Chatbots: 20 (69%) responses scored below 3 on a scale of 1 to 5, reflecting little to no experience in using chatbots to obtain medical information.

Knowledge of AI Tools in Clinical Settings: 23 (79%) responses also scored below 3, suggesting limited knowledge of specific AI applications in medicine.

**Figure 1:**
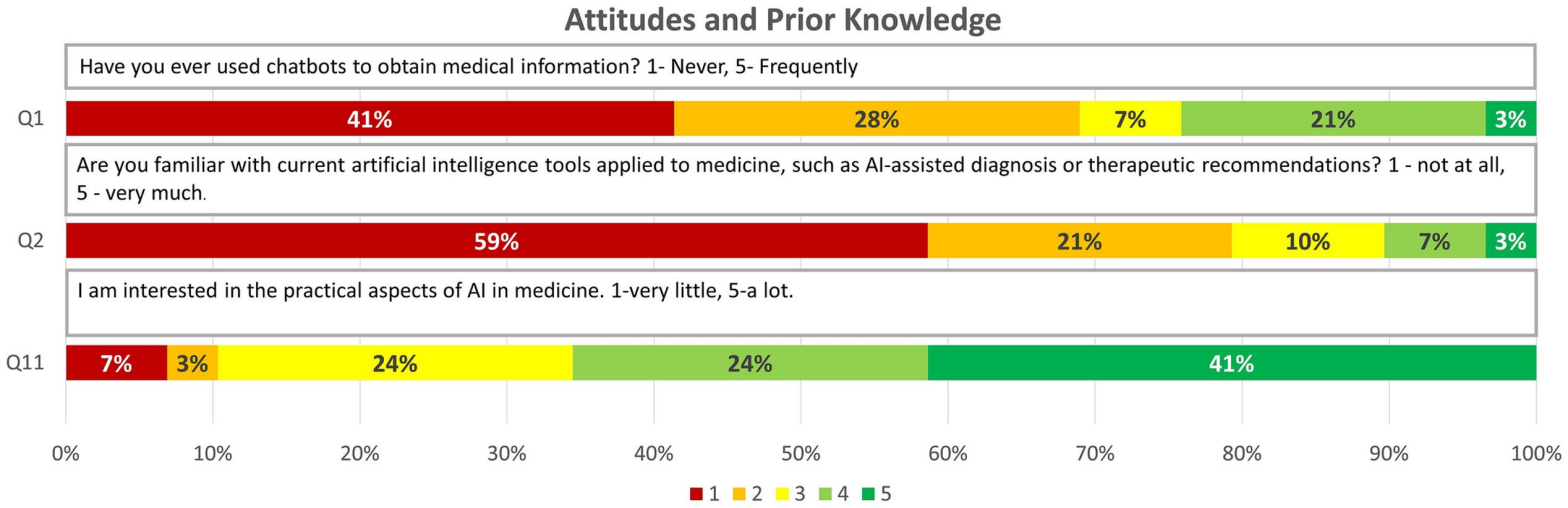
Graphic representation of the answers obtained for the questions on “Attitudes and Prior knowledge” through relative frequencies (1, 2, 11).

Despite this lack of familiarity, a notable interest in the practical applications of AI in the medical field emerged, with 19 (66%) scores exceeding 3. This indicates a positive attitude towards acquiring knowledge and skills related to these tools (Figure 1).

### 3.2 Ethical perceptions

This section explored concerns regarding the reliability, biases, and ethical implications of using AI-based chatbots in healthcare settings. The results revealed a significant level of concern:

Ethics of Using Chatbots: 14 (48%) participants rated above 3, indicating concerns about the moral and deontological implications of integrating these tools into medical practice.

Potential Biases: 17 (59%) expressed concern (scores >3) about the existence of biases, suggesting that students are aware of the risk of partiality in the recommendations or information provided by AI tools.

**Figure 2:**
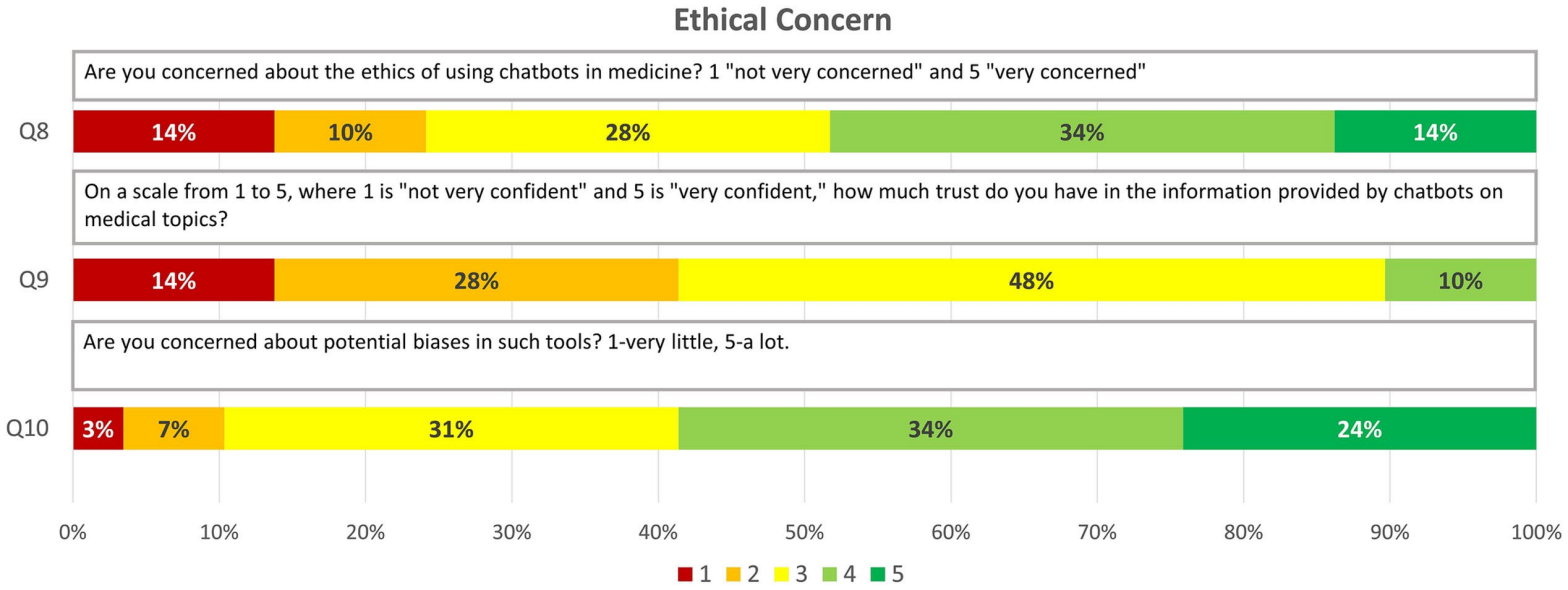
Graphic representation of the answers obtained for the questions on “Ethical concern” through relative frequencies (8–10).

Trust in Information Provided by Chatbots: Regarding the accuracy of the content supplied by chatbots, 17 (59%) scored ≥3, revealing moderate trust that is nevertheless tempered by the previously mentioned ethical doubts (Figure 2).

### 3.3 Future perspectives

The final section focused on opinions about the long-term impact of AI in medicine, including its effect on clinical practice, the training of future doctors, and the doctor-patient relationship. The findings suggest that students anticipate a significant change in their professional practice:

Positive Impact on Medicine: 100% of respondents rated ≥3, believing that AI can favourably transform medicine.

Educational Needs: 24 (83%) believe that doctors will require knowledge of AI to perform their duties effectively (≥3), and 26 (90%) consider that the medical curriculum should include AI (≥3), as well as practical content on its use (≥3).

Concerns about the Doctor-Patient Relationship: 25 (86%) perceive that the use of AI could negatively affect this relationship (≥3), and 19 (65%) believe it could contribute to the dehumanization of healthcare (≥3). Additionally, 22 (76%) fear the development of dependence on these tools (≥3).

Influence on Specialty Choice: 24 (83%) consider that the imposition of new technologies, such as AI, could influence their future decisions regarding medical specialization (≥3).

**Figure 3:**
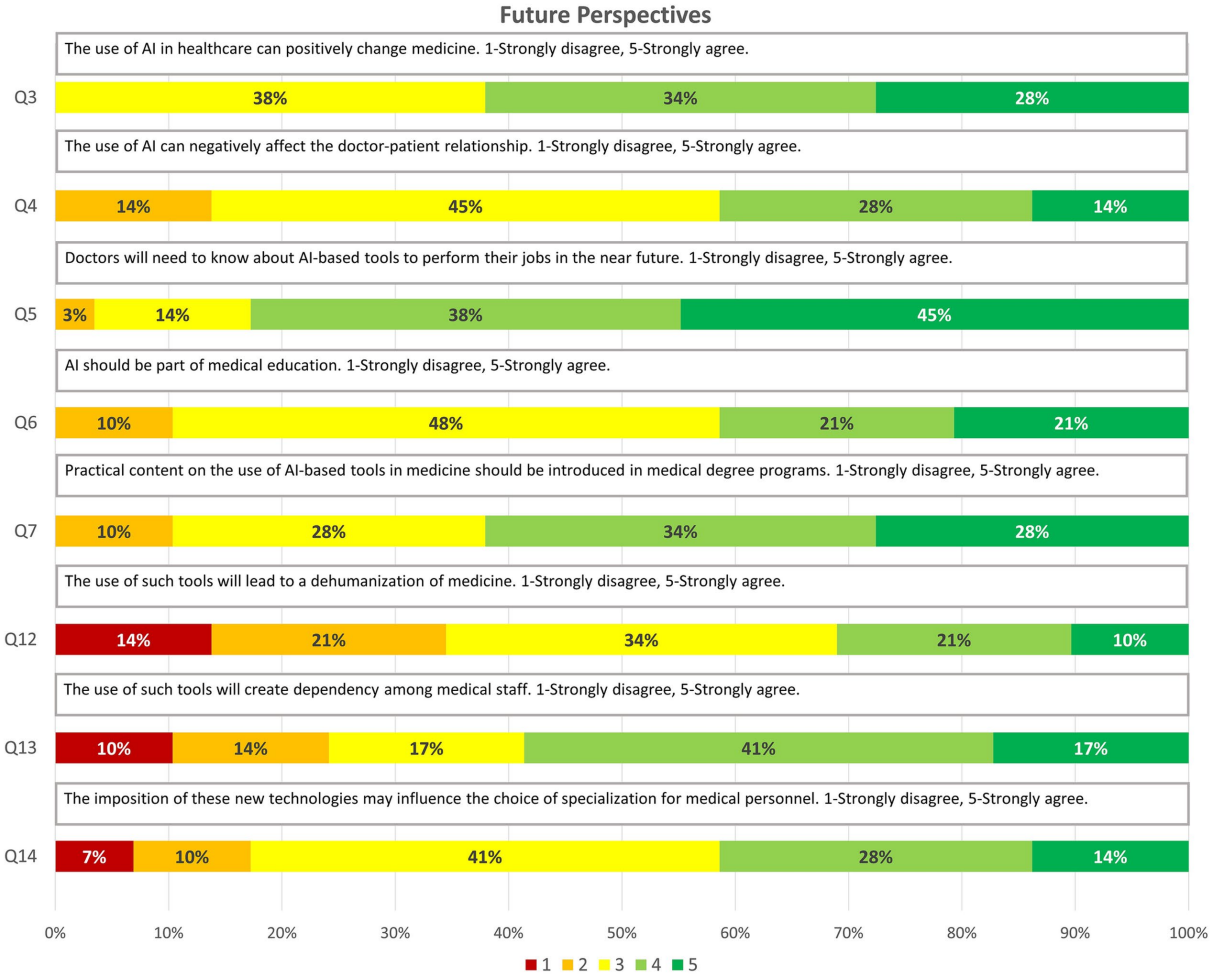
Graphic representation of the answers obtained for the questions on “Future perspectives” through relative frequencies (3–7, 12–14).

Overall, these results demonstrate that while students have limited prior contact with AI tools, they show a growing interest in learning and integrating them. They recognize the potential of AI to transform medicine and medical education but remain cautious about the ethical and human implications of its implementation. These perceptions, aligned with the study’s objective, provide an initial perspective on the educational needs, ethical concerns, and expectations of future healthcare professionals in the face of the increasing presence of AI in the health sector (Figure 3).

## 4 Discussion

The results obtained are consistent with the academic characteristics and formative stage of our sample of 29 medical students. These students, who have already completed Medical Ethics coursework and are concurrently engaging in clinical practices alongside theoretical subjects, represent an ideal profile for capturing how future healthcare professionals perceive the integration of Artificial Intelligence (AI) tools into their medical activities. Additionally, the non-mandatory nature of theoretical seminar attendance at this stage, combined with the documented absenteeism phenomenon in health sciences (20), reinforces the relevance of this sample as a study group.

This teaching improvement project aimed to explore the level of knowledge, ethical perceptions, and future perspectives of medical students regarding the use of AI tools in the healthcare field, specifically the employment of chatbots. Despite their limited direct experience with AI, the findings indicate that students are aware of the inherent ethical challenges of these technologies while recognizing the importance of acquiring competencies in this area for their future professional practice.

### 4.1 Attitudes and prior knowledge

The limited prior use of chatbots to obtain medical information aligns with trends described in the literature (21), indicating that these tools have not yet been widely incorporated into students’ routine information-seeking practices. This lack of familiarity suggests the need for specific educational interventions that increase exposure to AI and enhance understanding of its applications (22). Nevertheless, the positive disposition towards learning these technologies reflects an open field for curricular development.

### 4.2 Ethical challenges

The identification of ethical concerns by the students constitutes one of the most significant findings of this study, highlighting an area that warrants deeper attention. Participants expressed concerns about the accuracy of information, the presence of biases, data confidentiality, and the moral implications of using chatbots in clinical practice. This sensitivity to ethical dilemmas aligns with literature that underscores the importance of addressing these issues in the integration of AI in healthcare (17, 23, 24).

Although students showed a certain degree of trust in the responses provided by chatbots, this trust is tempered by the previously mentioned ethical reservations. It is clear that the mere incorporation of AI tools is insufficient: it is imperative to establish solid ethical frameworks, well-defined guidelines, and training that goes beyond technical competencies. Including ethics modules focused on AI, case-based discussions, and dialogues with ethics and technology experts could foster a critical and responsible view of the use of these tools. In this way, future doctors can adopt balanced approaches, ensuring safe, equitable, and patient-centered applications.

### 4.3 Future perspectives

The students’ perspectives suggest that AI could facilitate collaboration between healthcare professionals and chatbots, potentially optimizing care in an increasingly complex clinical environment (1). The nearly unanimous conviction that knowledge of these tools will be essential in their careers underscores the need to reform medical curricula, incorporating technological skills that prepare future professionals for a rapidly transforming care scenario (7, 25).

Furthermore, concerns about the risk of dehumanizing care, potential technological dependence, or the influence of AI on specialty choice should not be overlooked. These warnings highlight the importance of balancing technological literacy with the development of humanistic, ethical, and communication competencies. Extending these training strategies to other health science degrees will promote teamwork and a comprehensive approach to AI usage.

### 4.4 Limitations

Although this project provides valuable preliminary findings, it is important to acknowledge several limitations that affect the generalizability and robustness of the results. Firstly, the sample size was small, and participation was not mandatory, which not only impedes the representativeness of the general population of medical students but also introduces a non-response bias: those students who chose not to participate might hold different perceptions or attitudes regarding AI in education. Secondly, the study was conducted within the specific context of a seminar focused on AI, so the perceptions gathered could be influenced by the educational intervention itself, generating a potential acquiescence bias toward the presented environment.

Additionally, although the questionnaire used underwent a second iteration following a pilot with 14 participants and was agreed upon with expert educators, it lacks a formal psychometric validation process. The absence of objective questions that assess the actual level of knowledge limits the ability to contrast subjective perceptions with more direct indicators, and the simplicity of the instrument may not capture the real complexity of the perceptions, attitudes, and contextual factors that influence the use of AI in medical training environments.

To address these limitations, future research should consider using larger, more diverse samples with higher response rates to enhance representativeness and statistical power. It would also be advisable to evaluate the effectiveness of AI educational initiatives in different training contexts and over longer periods, as well as to refine and validate the questionnaire through rigorous psychometric analyses, incorporate objective questions, and encompass broader contextual factors. In this way, the conclusions drawn would be more robust, applicable, and generalizable to a wider range of medical education settings.

## 5 Conclusion

This teaching improvement project, aimed at describing the knowledge, attitudes, and perspectives of medical students regarding the application of AI and the use of chatbots in the healthcare field, revealed that participants are not significantly exposed to these tools nor are they a regular part of their academic or clinical routines. Despite this limited familiarity, they demonstrated a moderate awareness of the ethical challenges involved in incorporating AI into medical practice, reflecting an emerging sensitivity to the moral and deontological implications of these technologies.

At the same time, a marked optimism regarding the future adoption of AI-based tools was evident, as all students recognized the need to acquire knowledge in this area to perform effectively as healthcare professionals. This combination of ethical concerns and positive expectations underscores the importance of integrating specific AI-related educational content into medical education, enabling future doctors to use these tools effectively, thoughtfully, and responsibly.

Ultimately, the need to strengthen AI training within the medical curriculum not only responds to the growing presence of these technologies in healthcare delivery but also addresses the urgency of preparing tomorrow’s physicians to leverage the opportunities offered by AI while resolving the complex ethical implications associated with its implementation.

## Data Availability

The raw data supporting the conclusions of this article will be made available by the authors, without undue reservation.
